# Cotard’s Syndrome in a Patient With Paranoid Schizophrenia: A Case Report of a Type I Presentation

**DOI:** 10.7759/cureus.87731

**Published:** 2025-07-11

**Authors:** Mariam Tsuladze, Khatia Sulaberidze

**Affiliations:** 1 Psychiatry, Tbilisi State Medical University, Mental Health Centre, Tbilisi, GEO; 2 Psychiatry, Mental Health Centre, Tbilisi, GEO

**Keywords:** cotard's delusion, cotard syndrome, delusions of nonexistence, nihilistic delusion, paranoid schizophrenia

## Abstract

This report presents a rare case of Cotard's syndrome in a middle-aged woman with paranoid schizophrenia. The patient developed symptoms characterized by Cotard's syndrome in the psychiatric ward after several days of treatment. Our patient's presentation was not typical: she had a nihilistic delusion but lacked depressive symptoms. The patient was successfully treated with haloperidol, diazepam, and levomepromazine.

This case demonstrates the importance of identifying Cotard’s condition in non-depressed, schizophrenic individuals, as prompt management can improve outcomes.

## Introduction

Cotard's syndrome, also known as walking corpse syndrome, is a rare condition [1-3]. It is characterized by the nihilistic delusional belief that one's body, organs, or even existence has been destroyed or is nonexistent [2,4-6]. Cotard's syndrome was first described by Jules Cotard. He described it as anxious melancholia with ideas of damnation or rejection, insensitivity to pain, delusions of nonexistence concerning one’s own body, and delusions of immortality [2]. According to DSM-5, Cotard is not an independent diagnosis but rather a manifestation of an underlying disorder [7]. It can be part of different medical, neurological, and psychiatric diseases, including schizophrenia and other psychotic disorders [2,6,7]. 

Several treatment options have been described, including antidepressants, antipsychotics, and mood stabilizers [1,2]. Other than pharmacological therapy, electroconvulsive therapy (ECT) is also beneficial [1,2,4,7].

Our case describes a rare occurrence of Cotard's syndrome in a patient with paranoid schizophrenia and its successful treatment with haloperidol, diazepam, and levomepromazine.

## Case presentation

A 50-year-old woman with paranoid schizophrenia was admitted to a psychiatric facility due to worsening religious-type visual hallucinations, paranoid delusions, and refusal to eat after discontinuing her prescribed antipsychotic medication, but she couldn't recall when she had stopped taking it. She believed that a close family member was possessed by demons, which caused interpersonal conflict.

The patient had no family history of mental illness. The first developmental milestones fell within the normal range. She maintained average academic performance throughout her studies. In early adulthood, she dropped out of university, got married, and had children. Following her childbirth, the patient developed a persistent delusion that she had given birth to twins, with one of the infants allegedly concealed by the hospital. This conviction caused a conflict at home.

The patient had been unemployed for several years, had difficulties with daily tasks, but demonstrated occasional self-care abilities. She lived with and was cared for by her mother. Her mental history included multiple previous inpatient hospitalizations. She had been receiving regular follow-up care at a mental outpatient clinic for paranoid schizophrenia, including therapy with risperidone and trihexyphenidyl.

During the mental status examination, the patient was well-groomed, had good personal hygiene, displayed appropriate eye contact with the psychiatrist, and exhibited cooperative yet slightly guarded conduct. Her facial expression was somewhat inexpressive. The speech was spontaneous but delayed in response. The mood was depressed. The affect was blunted and poorly reactive. The thought process was paralogical. The reasoning was circumstantial, with episodes of over-elaboration (resonating). She demonstrated paranoid ideation and reported visual hallucinations. Although the patient lacked insight into her condition, she consented to treatment but refused oral medications.

Upon admission, the patient started receiving injectable medications: haloperidol, diazepam, and levomepromazine. All her medications are listed in Table 1.

**Table 1:** Intramuscular medication administration schedule

Time	Medication	Dose	Route	Frequency
10:00	Spridol (Haloperidol)	1.0 mL (1 amp)	IM	Twice daily (10:00, 16:00)
10:00	Diazepam	2.0 mL (1 amp)	IM	Twice daily (10:00, 16:00)
16:00	Spridol (Haloperidol)	1.0 mL (1 amp)	IM	
16:00	Diazepam	2.0 mL (1 amp)	IM	
21:00	Tizercin (Levomepromazine)	1.0 mL (1 amp)	IM	Once daily (21:00)

Within the first week of treatment, the patient started showing symptoms characteristic of Cotard's syndrome, including delusions of being dead. Time and again, she claimed to be "a walking corpse" and to be "losing her soul." She kept saying: "A saint came into my body right at death. I am physically dead; just the saint's soul is alive in me.” She also expressed hypochondriac and nihilistic delusions, such as "My intestines are now severed." "My stomach ought not to work." "I cannot eat anything-I cannot digest anything." "I have no internal organs." "I am the walking dead."

The patient continued to adhere to the treatment plan despite experiencing strong delusional beliefs. After a few weeks of treatment, she resumed eating, appeared calmer and more organized during her hospitalization, her mood improved, and she resumed basic self-care activities such as grooming and dressing herself. Two months after admission, she was administered a 2 mL IM injection of diazepam and 50 mg/mL haloperidol decanoate and was discharged with no remaining delusions or hallucinations, indicating a significant improvement in her condition.

## Discussion

This case illustrates Cotard's syndrome in a patient with paranoid schizophrenia. When the patient checked in at the hospital, she had nihilistic and hypochondriac delusions along with delusions of possession. Only after several days of treatment did these symptoms become apparent.

The patient's symptoms indicate Cotard syndrome Type I, which is characterized by prominent nihilistic delusions without accompanying depressive symptoms, in contrast to Type II, where such delusions occur in the context of severe mood disturbance, particularly psychotic depression, according to Berrios and Luque (1995) [2,3,5,7,8].

Furthermore, Grover et al. (2014) noted that Cotard's patients frequently develop self-starvation as a result of nihilistic delusions, which was also the case with our patient, who refused to eat [3]. This shows a significant danger of Cotard's syndrome, also known as "walking corpse syndrome," due to the neglect of eating, mainly because patients believe they are "already dead" [3].

Morgado et al. reported on a case study of a 42-year-old man with chronic paranoid schizophrenia and a history of substance use who developed Cotard's delusions despite maintaining an overall stable psychotic condition. Similar to our patient, he experienced delusions of nonexistence (e.g., "my body parts are gone") without any associated depression. Treatment with the antipsychotic resulted in complete remission of both psychotic and nihilistic delusions, which was the case with our patient as well [9]. Both studies emphasize the effectiveness of antipsychotic medications in managing Cotard's syndrome [9].

When comparing our patient with others in the literature, not only similarities but also differences are revealed. Our patient is a middle-aged female, which is a standard demographic profile for patients with Cotard's syndrome (median age around 52) [1,4,10]. However, in contrast to most cases where patients are depressed, she showed no mood symptoms and had a psychotic/paranoid profile. Her delusions had noticeable hypochondriacal marks, a common historical description for such presentations. Finally, her condition emerged in the context of established schizophrenia, an association that has been documented as extremely rare and may involve a higher self-risk [6,9].

## Conclusions

Despite the fact that Cotard's syndrome is not classified as a separate disorder in DSM-5, this case demonstrates the importance of identifying and managing the symptoms. Early recognition is vital for patients who refuse to eat or self-harm because of delusions.

In addition, it is important to note that Cotard's disorder can be seen not only in psychiatric disorders but also in neurological and medical conditions. Even though the occurrence of Cotard's disorder is very rare, we have different treatment options that have proven to be successful.
